# Refractory Thigh Pain Revealing Femoral Neuritis in Antineutrophil Cytoplasmic Antibody-Associated Vasculitis: A Case Report

**DOI:** 10.7759/cureus.115508

**Published:** 2026-08-31

**Authors:** Jerry Luo, Timothy R Cushing, Jessie D Bjella, Daun Chung, Adam H Saby

**Affiliations:** 1 Medicine, UCLA Health, Los Angeles, USA

**Keywords:** antineutrophil cytoplasmic antibody (anca)-associated vasculitis (aav), femoral neuritis, intractable leg pain, lower extremity weakness, neuropathic pain

## Abstract

Peripheral nervous system involvement is a possible manifestation of antineutrophil cytoplasmic antibody (ANCA)-associated vasculitis (AAV). We present a case of a 48-year-old female patient with known AAV presenting with refractory left thigh pain. She underwent extensive workup for musculoskeletal, spinal, and vascular causes of her pain that were all unrevealing. Physical examination localized her pain to the femoral nerve, and magnetic resonance imaging (MRI) of the pelvis with neurography revealed findings consistent with femoral neuritis. She was promptly started on immunosuppressive treatment, which resulted in dramatic improvement in her pain. This case demonstrates that vasculitic neuropathy may present as a painful proximal mononeuropathy. Neurologic localization and targeted magnetic resonance neurography should be considered when conventional evaluation fails to explain intractable focal extremity pain in a patient with systemic vasculitis.

## Introduction

Antineutrophil cytoplasmic antibody (ANCA)-associated vasculitides (AAV) are a group of systemic disorders characterized by small-vessel inflammation with variable involvement of multiple organ systems [1]. The three major variants of AAV are granulomatosis with polyangiitis (GPA), microscopic polyangiitis (MPA), and eosinophilic granulomatosis with polyangiitis (EGPA) [2]. Peripheral nervous system involvement in AAV can cause significant pain, weakness, numbness, and functional impairment [3]. The most common pattern of vasculitic peripheral neuropathy is mononeuritis multiplex, which involves multiple peripheral nerves [4, 5]. Here we present a case of isolated femoral neuritis associated with AAV that presented as refractory thigh pain. Documentation of this case expands the recognized clinical spectrum of vasculitic neuropathies and may promote earlier diagnosis of inflammatory nerve disease in patients with unexplained focal limb pain. 

## Case presentation

A 48-year-old female patient with ANCA-associated vasculitis, well-controlled type 2 diabetes mellitus with a most recent hemoglobin A1c of 4.9% (normal: <5.7%), and end-stage renal disease on hemodialysis presented with severe left lower extremity pain. She initially presented to the emergency department in July 2025 with two weeks of progressively worsening anterior left thigh pain that radiated into the left lower quadrant of the abdomen without any inciting event such as trauma or overuse. The quality of pain was described as aching and sharp, and she denied neuropathic features, including any electric, burning, or cold character. Her pain was refractory to all medications administered in the inpatient setting, including gabapentin, methocarbamol, intravenous hydromorphone, and ketamine infusion. 

On examination, vital signs were within normal limits. There was tenderness to palpation throughout the patient’s left anterolateral thigh. She tolerated both passive hip and knee range of motion without exacerbation of pain. The motor exam, which may have been confounded by pain, had noticeably diminished strength in the left hip flexors (4-/5), quadriceps (4/5), and knee flexors (4/5). Excluding these three muscle groups, strength was 5/5 in the bilateral lower extremities. Reflexes were symmetric in the bilateral lower extremities, and the sensory exam was normal. On special testing, she had a positive straight leg raise and femoral nerve stretch test on the left. Although these provocative tests were nonspecific, the combined straight leg raise, femoral nerve stretch test, quadriceps weakness, and anterior thigh pain raised concern for an L2-L4 radiculopathy, lumbar plexopathy, or femoral neuropathy. These three conditions could not be distinguished from one another based on examination alone, as a lesion at any of these levels could potentially produce quadriceps weakness and anterior thigh pain. The knee flexor weakness was not anatomically consistent with an isolated femoral nerve lesion, as the hamstrings are innervated by the sciatic nerve. Given the patient's severe pain during motor testing, this finding was felt to likely represent pain-limited weakness rather than a true neurological deficit, and this finding was not used to support localization to the femoral nerve.

Laboratory workup, which included obtaining an ANCA panel, showed that all assays were negative, which included the following: cytoplasmic-ANCA (C-ANCA) of <1:20 titer (normal: <1:20), perinuclear-ANCA (P-ANCA) of <1:20 titer (normal: <1:20), proteinase-3 antibody of <20.0 CU (normal: <20.0 CU), and myeloperoxidase antibody of <20.0 CU (normal: <20.0 CU).

The patient subsequently underwent exhaustive magnetic resonance imaging (MRI) and computed tomography (CT) of the spine and left lower extremity. MRI of the lumbar spine only demonstrated a mild disc bulge at L5-S1 but otherwise no significant disc protrusion, spinal canal stenosis, or neural foraminal stenosis that would explain the patient's symptoms (Figure 1). An MRI of the left hip demonstrated a chronic labral tear as well as partial tearing of the gluteus minimus and medius at the insertion; however, these findings were not consistent with the patient's pain complaints or physical exam findings. MRI of the left femur was unremarkable. CT of the abdomen and pelvis did not demonstrate any bleed, abscess, or mass. A CT angiogram (CTA) of the left lower extremity demonstrated severe peripheral arterial disease (PAD). The vascular surgery service was subsequently consulted and felt that PAD was unlikely to be responsible for the patient's pain complaints given the acuity of symptoms, as PAD is chronic in nature, as well as a lack of claudication symptoms. 

**Figure 1:**
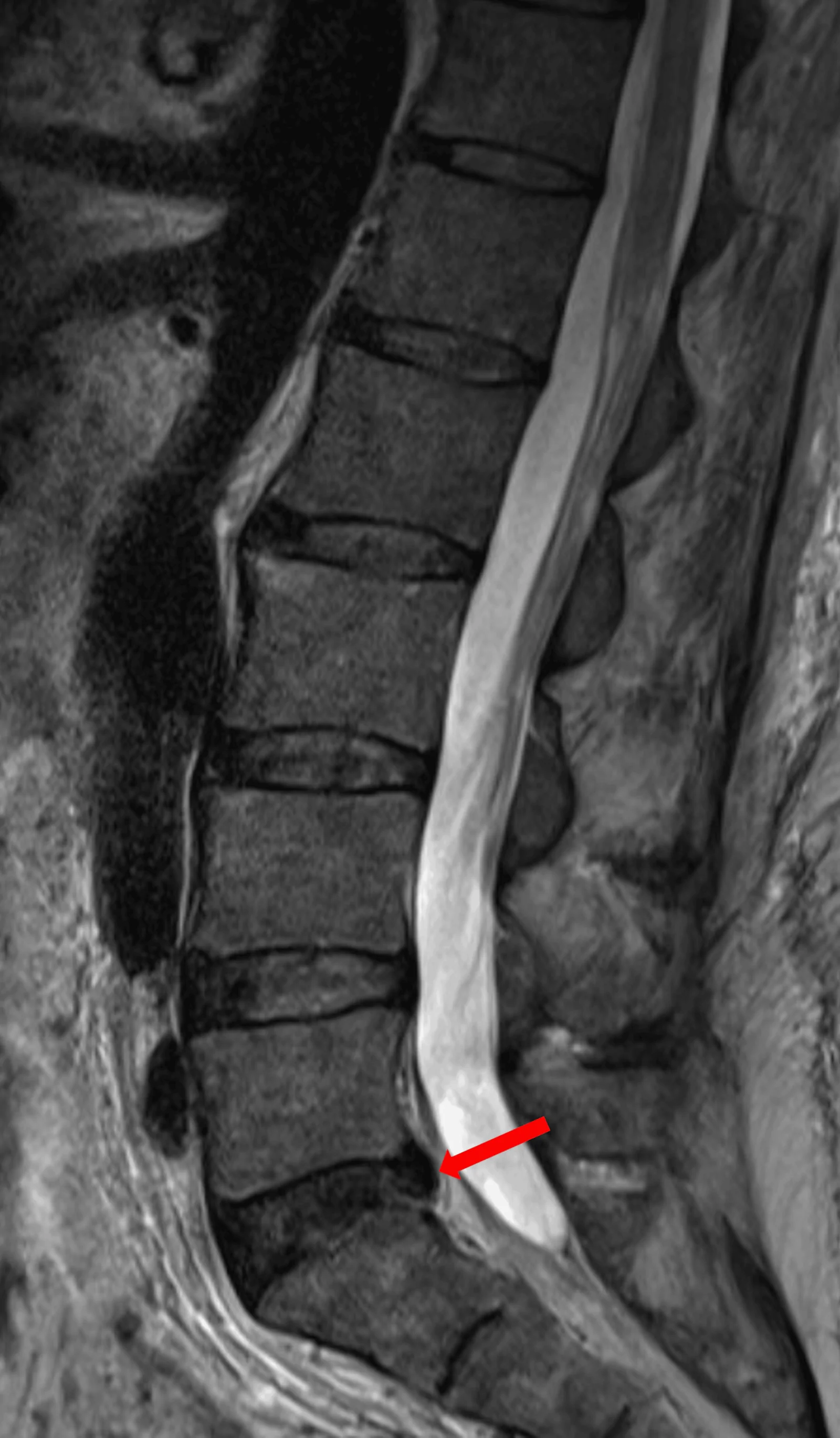
Sagittal view of the lumbar spine MRI without contrast. Mild disc bulge at L5-S1 is noted (red arrow).

On the 18^th^ day of admission, after extensive medical imaging, an MRI of the pelvis with and without contrast and with nerve imaging was obtained to rule out a possible femoral neuropathy or lumbar plexopathy. Although the patient’s pain lacked classic neuropathic pain features, the distribution of pain and weakness localized relatively well to the femoral nerve distribution. An MRI of the pelvis ultimately revealed asymmetric T2 hyperintensity and enhancement along the left femoral nerve, findings compatible with acute left femoral neuritis (Figure 2).

**Figure 2:**
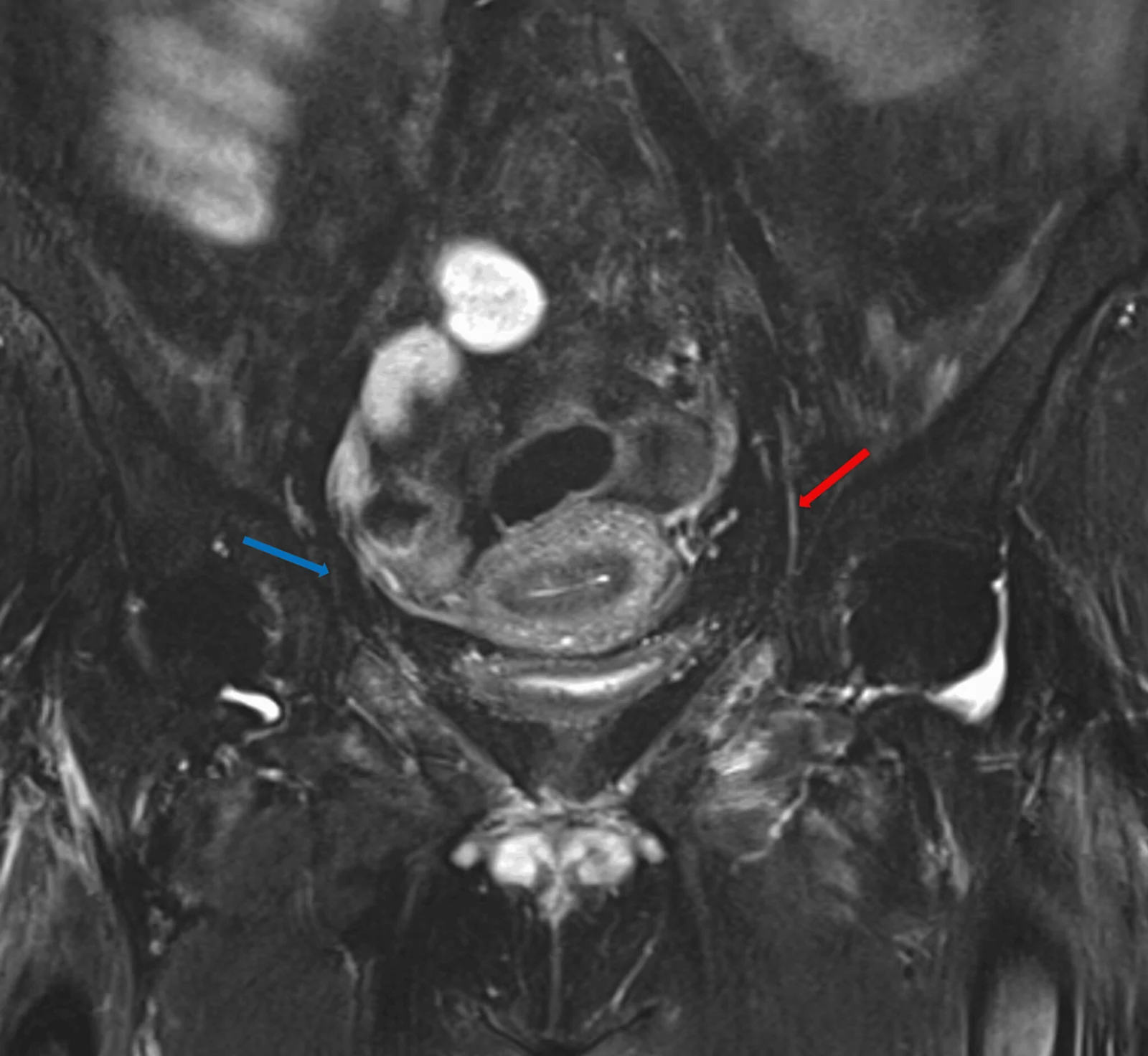
Coronal view of the pelvic MRI with MR neurography. Asymmetric T2 hyperintensity and enhancement of the left femoral nerve are seen (red arrow). The right femoral nerve appears to be normal (blue arrow).

Following the pelvis MRI results, rheumatology recommended that the patient be started on pulse-dose steroids with 1000 mg of intravenous methylprednisolone for three days, followed by five days of 1 mg/kg (60 mg) of intravenous methylprednisolone daily. Additionally, she completed two doses of rituximab (375 mg/m² body-surface area), with the first dose given inpatient and the second dose given one week later in the outpatient setting. The patient reported substantial relief of pain after the first dose of intravenous steroids and was quickly weaned off all intravenous pain medications and successfully discharged home. At the eight-month and eleven-month outpatient follow-up appointments, she reported resolution of thigh pain and was in the process of tapering off of oral steroids without recurrence of pain.

It is important to note that electrodiagnostic testing and nerve biopsy were not conducted, and as such, the diagnosis of AAV femoral neuritis must be considered presumptive. If electrodiagnostics were completed, findings supportive of femoral neuropathy could have included active denervation in femoral nerve-innervated muscles and reduced amplitudes in the femoral compound muscle action potential and saphenous sensory nerve action potential [6]. Given the context of a previously established AAV diagnosis, rapid improvement of symptoms following immunosuppressive therapy, and absence of an otherwise compressive lesion after extensive imaging workup, AAV femoral neuritis was considered the most likely diagnosis.

## Discussion

Peripheral nervous system involvement is a variable feature of AAV, with an estimated prevalence of 58% in MPA, 17% in GPA, and 77% in EGPA [7-9]. In vasculitic neuropathy, vasa nervorum inflammation leads to both focal and multifocal axonal ischemia [10]. In the ANCA-associated vasculitides, this is a sequential process that starts with ANCA-mediated activation of neutrophils, triggering release of inflammatory cytokines, lytic enzymes, and reactive oxygen species, which damage endothelial cells [11].

Nervous system involvement in AAV classically presents as mononeuritis multiplex [4]. Mononeuritis multiplex is a clinical pattern by which multiple, non-contiguous peripheral nerves are serially or simultaneously injured, resulting in asymmetric, multifocal sensorimotor deficits. This typically occurs over days to weeks rather than simultaneously, as each nerve infarction is a discrete event [5]. Depending on the time of presentation, if multiple peripheral nerves have already been injured, mononeuritis multiplex may mimic a symmetric polyneuropathy [12].

The Diagnostic and Classification criteria for primary systemic VASculitis (DCVAS) study, which contains the largest prospectively studied AAV patient population, demonstrated that 47% of patients with electrodiagnostically confirmed peripheral neuropathy presented as mononeuritis multiplex. The DCVAS study did not, however, report on the specific percentage of isolated mononeuropathies [4]. Further literature review corroborates mononeuritis multiplex as the most common clinical pattern of peripheral neuropathy, with some studies demonstrating an occurrence of over 70% [8,13]. The peroneal or tibial nerves of the lower extremities are most commonly affected first [14]. Femoral nerve involvement appears rare and has not been well documented in previous literature [15].

Our presented case has several features that are atypical. The patient presented with an isolated mononeuropathy of the femoral nerve, and sequential nerve involvement was not observed. It is feasible, however, that she may have developed mononeuritis multiplex if pulse steroids were not given in a timely manner. Although the distribution of pain lacked neuropathic features and weakness clinically localized to the femoral nerve and pelvic MRI supported this, electrodiagnostic studies were ultimately not performed, and the diagnosis was not electrophysiologically confirmed. As such, the diagnosis of isolated femoral neuritis remains presumptive and should be interpreted with appropriate caution. Clinicians should maintain a broad differential in patients with unexplained refractory pain, especially when the clinical course is progressive and inconsistent with typical features of mononeuritis multiplex. AAV neuropathy can present as a painful, isolated proximal mononeuropathy, and the absence of neuropathic qualities or early multifocal involvement does not exclude an inflammatory neuropathy.

## Conclusions

This case describes an uncommon presentation of presumptive isolated femoral neuritis causing severe intractable lower extremity pain in the setting of known AAV. The presenting symptoms of the patient were initially more suspicious for a more common musculoskeletal condition such as lumbar radiculopathy or hip pathology, which prevented early diagnosis of vasculitic neuropathy. Although the clinical and imaging findings as well as rapid response to immunosuppressive therapy supported this diagnosis, the absence of electrodiagnostic confirmation limits diagnostic certainty. Increased recognition of this presentation may reduce irreversible systemic complications and progressive neurological deficits by resulting in earlier initiation of immunosuppressive therapy.
